# An increase in galectin-3 causes cellular unresponsiveness to IFN-γ-induced signal transduction and growth inhibition in gastric cancer cells

**DOI:** 10.18632/oncotarget.7750

**Published:** 2016-02-26

**Authors:** Po-Chun Tseng, Chia-Ling Chen, Yan-Shen Shan, Chiou-Feng Lin

**Affiliations:** ^1^ Institute of Clinical Medicine, College of Medicine, National Cheng Kung University, Tainan 701, Taiwan; ^2^ Translational Medicine Center, Taipei Medical University, Taipei 110, Taiwan; ^3^ Department of Surgery, College of Medicine, National Cheng Kung University, Tainan 701, Taiwan; ^4^ Graduate Institute of Medical Sciences, College of Medicine, Taipei Medical University, Taipei 110, Taiwan; ^5^ Department of Microbiology and Immunology, School of Medicine, College of Medicine, Taipei Medical University, Taipei 110, Taiwan

**Keywords:** IFN-γ, Galectin-3, AKT, GSK-3β, SHP2

## Abstract

Glycogen synthase kinase (GSK)-3β facilitates interferon (IFN)-γ signaling by inhibiting Src homology-2 domain-containing phosphatase (SHP) 2. Mutated phosphoinositide 3-kinase (PI3K) and phosphatase and tensin homolog (PTEN) cause AKT activation and GSK-3β inactivation to induce SHP2-activated cellular unresponsiveness to IFN-γ in human gastric cancer AGS cells. This study investigated the potential role of galectin-3, which acts upstream of AKT/GSK-3β/SHP2, in gastric cancer cells. Increasing or decreasing galectin-3 altered IFN-γ signaling. Following cisplatin-induced galectin-3 upregulation, surviving cells showed cellular unresponsiveness to IFN-γ. Galectin-3 induced IFN-γ resistance independent of its extracellular β-galactoside-binding activity. Galectin-3 expression was not regulated by PI3K activation or by a decrease in PTEN. Increased galectin-3 may cause GSK-3β inactivation and SHP2 activation by promoting PDK1-induced AKT phosphorylation at a threonine residue. Overexpression of AKT, inactive GSK-3β^R96A^, SHP2, or active SHP2^D61A^ caused cellular unresponsiveness to IFN-γ in IFN-γ-sensitive MKN45 cells. IFN-γ-induced growth inhibition and apoptosis in AGS cells were observed until galectin-3 expression was downregulated. These results demonstrate that an increase in galectin-3 facilitates AKT/GSK-3β/SHP2 signaling, causing cellular unresponsiveness to IFN-γ.

## INTRODUCTION

Gastric adenocarcinoma is caused by a variety of carcinogenic stimuli, including *Helicobacter pylori* infection, tobacco, dietary factors, and host gene polymorphisms [1–3]. Studies showed that oncogenic activation (including activation of phosphoinositide 3-kinase (PI3K)/AKT, Ras/Raf/mitogen-activated protein kinase kinase (MEK)/extracellular signal-regulated kinase (ERK) and growth factor receptors), inactivation of tumor suppressors (e.g., p53 and adenomatous polyposis coli mutations), and reduced phosphatase and tensin homolog (PTEN) and runt-related transcription factor 3 expression levels are involved in gastric tumor growth and survival [4, 5]. Additionally, gastric cancers may require escape from immune surveillance, thereby developing advanced survival strategies [6, 7]. However, the crosstalk between oncogenic processes and immune escape strategies is undocumented.

Galectin-3, one of the galectin family proteins that are defined by their binding specificity for β-galactoside sugars, has a chimeric structure containing one conserved carbohydrate-recognition domain and a long non-lectin domain [8]. Extracellular galectin-3 can bind to glycoproteins and glycolipids in cell membranes to control the cell cycle and apoptosis [9]. In contrast, cytoplasmic galectin-3 can bind to Bcl-2 to promote cell survival and inhibit apoptosis [10]. Galectin-3 is overexpressed in several human cancers, including gastric, colon, and pancreatic cancers [11–13]. Furthermore, oncogenic galectin-3 may induce cellular transformation through the Ras and PI3K/AKT signaling pathways [14, 15]. In gastric cancers, galectin-3 increases cell motility by upregulating fascin-1, protease-activated receptor-1, and matrix metalloproteinase-1 expression levels [16, 17]. A galectin-3 germline variant induces nuclear accumulation and activation of β-catenin [18]. Therefore, decreasing galectin-3 can serve as a strategy against gastric tumorigenesis.

For cancer immunosurveillance, T/NK cells confer anticancer immunity by secreting several cytotoxic molecules, including interferon (IFN)-γ, perforin, granzymes, CD95 ligand, and TRAIL [7, 19, 20]. Immune IFN-γ exhibits anticancer activities by upregulating the expression levels of tumor-suppressing factors, such as the Fas/Fas ligand, p53, caspases, and major histocompatibility complex (MHC) molecules, and by inducing cell growth inhibition and cytotoxicity [21–23]. Indeed, T/NK cell-derived IFN-γ attenuates cancer cell growth *in vitro* and *in vivo* [24–26]. Gastric cancers commonly show a decreased level of MHC I expression [27, 28], indicating an endogenous defect in IFN-γ signaling. Only a few reports have shown a defective response of MHC I expression in IFN-γ-resistant AGS cells [29, 30]; however, possible mechanisms of IFN-γ resistance remain unknown. To control IFN-γ-activated JAK2/signal transducer and activator of transcription (STAT)1 signaling, Src homology-2 domain-containing phosphatase (SHP)2 can dephosphorylate JAK2 and STAT1 to suppress IFN-γ signaling [23, 31–33]. We hypothesize that cancers may acquire aberrant SHP2 to avoid the immune defense of IFN-γ. We previously showed that glycogen synthase kinase (GSK)-3β facilitates IFN-γ-activated STAT1 by inhibiting SHP2 [34], and aberrant PI3K and a decrease in PTEN increase AKT activation and GSK-3β inactivation to cause SHP2-activated IFN-γ resistance in gastric cancer AGS cells [35]. In the present study, we investigated the crosstalk of galectin-3 with AKT/GSK-3β signaling and IFN-γ resistance in gastric cancer cells.

## RESULTS

### Increasing or decreasing galectin-3 expression changes IFN-γ signaling

**Figure 1:**
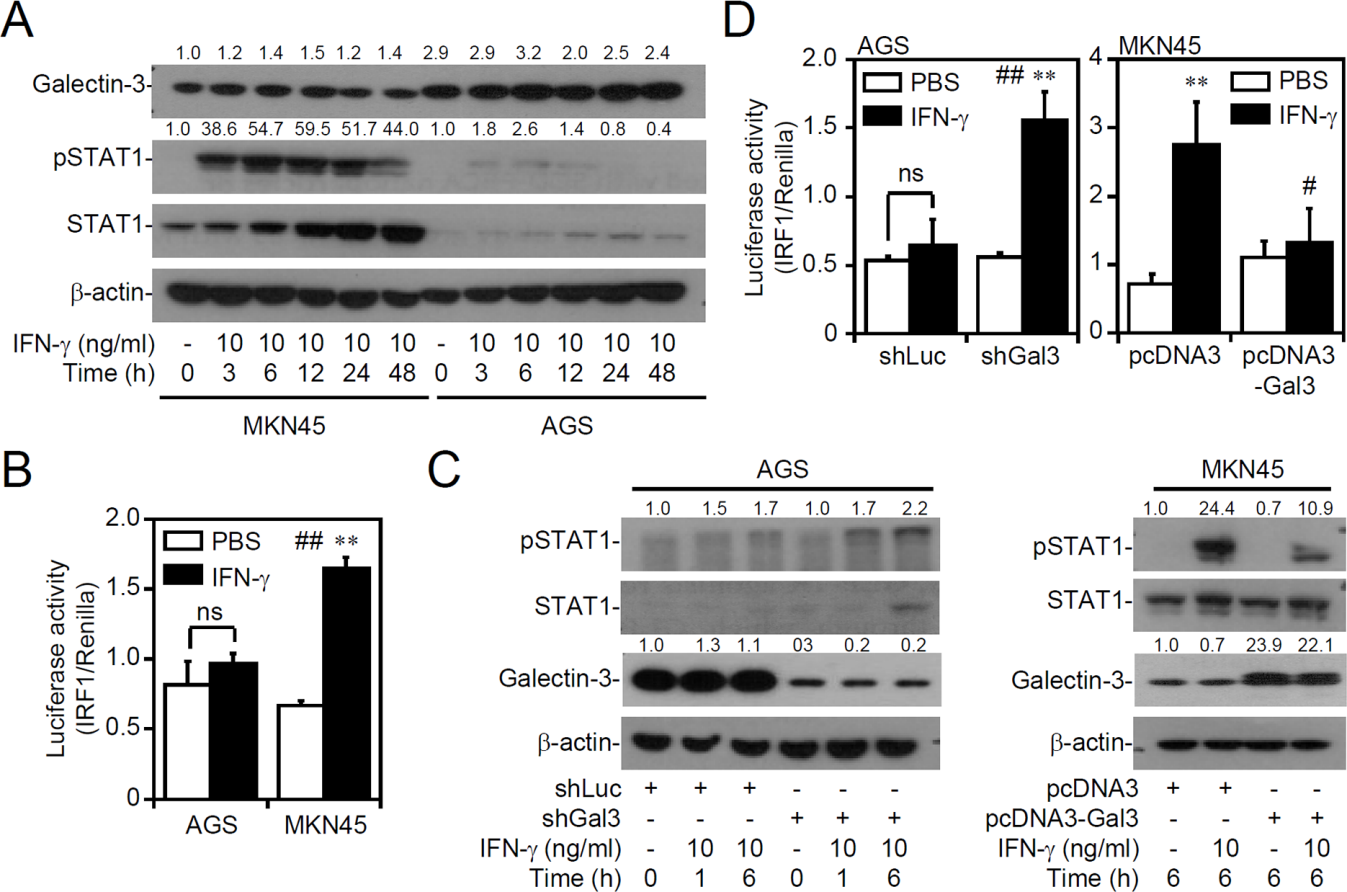
Decreasing or increasing galectin-3 expression interferes with interferon (IFN)-γ signaling Western blot of the indicated proteins (**A.** and **C.**) and detection of IRF1 transactivation using a luciferase reporter assay (**B.** and **D.**) in MKN45 (IFN-γ sensitive) and AGS (IFN-γ insensitive) cells treated with IFN-γ for the indicated times or in IFN-γ-treated AGS cells transfected with shRNA targeting luciferase (*shLuc*) and shRNA targeting galectin-3 (*shGal3*), and IFN-γ-treated MKN45 cells transfected with pcDNA3 and pcDNA3-Gal3. For Western blotting, β-actin was used as an internal control. A representative dataset from triplicate experiments is shown. For the luciferase reporter assay, the ratio of IRF1 to control Renilla is shown, and the data are presented as the mean ± SD from three independent experiments. ns, not significant. **p < 0.01 compared to PBS; ^#^*p* < 0.05 and ^##^*p* < 0.01 compared to the relative control.

We previously demonstrated that, compared to IFN-γ-sensitive MKN45 cells, AGS cells are resistant to IFN-γ-induced signaling and cell growth inhibition [35]. Because SHP2 can be activated by the PI3K/AKT-mediated pathway, aberrant expression of galectin-3, an oncogenic protein that acts upstream of AKT [14, 15], was next examined in gastric cancer cells. Western blotting showed an increased level of galectin-3 in IFN-γ-insensitive AGS cells accompanied by the generation of cellular unresponsiveness to IFN-γ-induced STAT1 phosphorylation at Tyr701 (Figure 1A) and IRF1 transactivation (Figure 1B). We next evaluated the effects of galectin-3 on IFN-γ signaling. In galectin-3-silenced AGS cells, IFN-γ ultimately induced STAT1 phosphorylation at Tyr701 (Figure 1C, left). In contrast, overexpression of galectin-3 in MKN45 cells inhibited STAT1 phosphorylation (Figure 1C, right). The IRF1 transactivation assay confirmed the different responses of IFN-γ signaling in galectin-3-silenced AGS cells (Figure 1D, top) and galectin-3-overexpressing MKN45 cells (Figure 1D, bottom). Furthermore, galectin-3 overexpression in THP1 and U937 cells was also resistant to IFN-γ-activated IRF1 (Supplementary Figure S1). Similarly, changes in galectin-3 expression in AGS and MKN45 cells did not increase or decrease the expression of IFNGR1 or IFNGR2 in those cells (Supplementary Figure S2). These results indicate that increasing or decreasing galectin-3 expression changes IFN-γ signaling.

### Increased galectin-3 in cells shows cellular unresponsiveness to IFN-γ-activated IRF1

**Figure 2:**
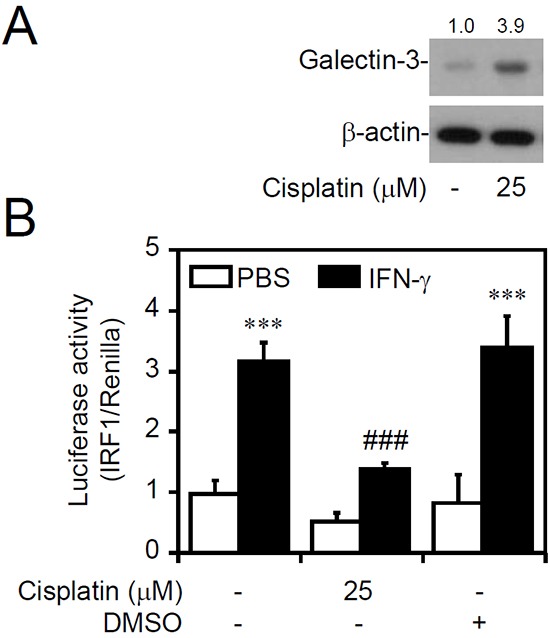
Inducible galectin-3 causes cellular insensitivity to interferon (IFN)-γ Western blot of the indicated proteins **A.** and detection of IRF1 transactivation using a luciferase reporter assay **B.** in MKN45 cells (IFN-γ sensitive) treated with cisplatin for 24 h, followed by IFN-γ treatment for 6 h. For Western blotting, β-actin was used as an internal control. A representative dataset from triplicate experiments is shown. Luciferase reporter assay showing the ratio of IRF1 to control Renilla, and the data are presented as the mean ± SD from three independent experiments. ***p < 0.001 compared to PBS; ^###^*p* < 0.001 compared to IFN-γ alone.

To further evaluate the effect of increased galectin-3 causing IFN-γ resistance, a natural inducible approach was utilized as previously described [36], and it showed that galectin-3 can be induced in surviving cells under cisplatin stimuli. Accordingly, treating human leukemia K562 cells with the chemotherapeutic agent cisplatin effectively increased galectin-3 expression (Figure 2A). The IRF1 transactivation assay demonstrated a significant blockade effect on IFN-γ-activated IRF1 in cisplatin-treated cells (Figure 2B). These findings suggest that the inducible expression of galectin-3 in cells shows cellular unresponsiveness to IFN-γ stimulation.

### The pharmacological inhibition of extracellular galectin-3 does not decrease IFN-γ resistance

**Figure 3:**
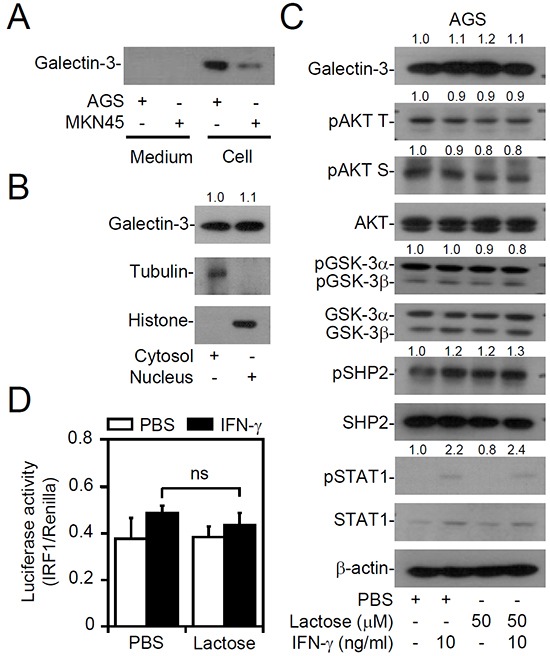
No extracellular galectin-3 is involved in interferon (IFN)-γ insensitivity of AGS cells Western blot of the indicated proteins in supernatants and cell lysates of AGS and MKN45 cells **A.**, in the cytosolic and nuclear fractions of AGS cells **B.**, and in AGS cells pretreated with lactose for 0.5 h, followed by IFN-γ treatment for another 6 h **C.** Tubulin and histone were respective markers for the cytosolic and nuclear fractions. **D.** A luciferase reporter assay detected IRF1 transactivation in AGS cells pretreated with lactose for 0.5 h, followed by IFN-γ treatment for another 6 h. For Western blotting, β-actin was used as an internal control. pAKT T, phospho-AKT at Thr308; pAKT S, phospho-AKT at Ser473. A representative dataset from triplicate experiments is shown. For the luciferase reporter assay, the ratio of IRF1 to control Renilla is shown, and the data are presented as the mean ± SD from three independent experiments. ns, not significant.

Galectin-3 is expressed within cells and is also expressed as a soluble protein via autocrine and paracrine routes [8]. To evaluate the potential role of extracellular galectin-3, we collected culture supernatants from AGS and MKN45 cells to detect protein expression. However, Western blot analysis detected no secreted galectin-3 expression in either AGS or MKN45 cells (Figure 3A). Further cytosolic/nuclear extraction studies in AGS cells showed that galectin-3 was expressed in both the cytosol and the nuclei (Figure 3B). To confirm inhibition of the effect of extracellular galectin-3, we applied exogenous lactose, which inhibits galectin-3 by inhibiting β-galactoside-binding activity. Western blot (Figure 3C) and IRF1 transactivation assays (Figure 3D) showed no changes in AKT/GSK-3β/SHP2 signaling, which is known to be a negative regulator of IFN-γ signaling [35], and IFN-γ-activated STAT1 and IRF1. Furthermore, treatment with modified citrus pectin [37], an antagonist specific to the galectin-3 carbohydrate-recognition domain, did not affect IFN-γ and AKT/GSK-3β/SHP2 signaling (Supplementary Figure S3). These results indicate that intracellular but not extracellular galectin-3 is vital for the cellular unresponsiveness to IFN-γ in AGS cells.

### IFN-γ-insensitive AGS cells show an increase in galectin-3 expression independent of deregulated PI3K and PTEN expression levels

**Figure 4:**
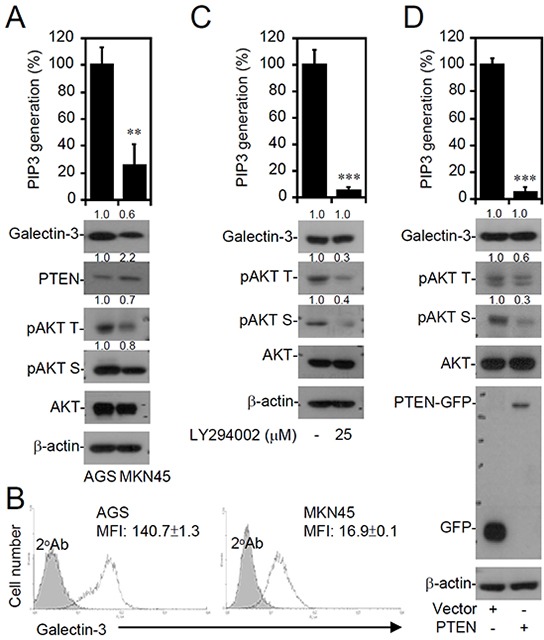
Increased galectin-3 expression correlates with PI3K-AKT activation and PTEN decrease in interferon (IFN)-γ-insensitive AGS cells A PIP3 MASS ELISA Kit assay and Western blot analysis detected PI3K activity and expressions of the indicated proteins, respectively, in untreated AGS (IFN-γ insensitive) and MKN45 (IFN-γ sensitive) cells **A.**, AGS cells treated with the PI3K inhibitor, LY294002, for 24 h **C.**, and AGS cells transfected with pcDNA3.1-GFP or pcDNA3.1-GFP-PTEN **D.** For kinase activity, data are the mean ± SD from three independent experiments. **p < 0.01 and ***p < 0.001 compared to the control. For Western blotting, β-actin was used as an internal control. pAKT T, phospho-AKT at Thr308; pAKT S, phospho-AKT at Ser473. A representative dataset from triplicate experiments is shown. **B.** A representative histogram of immunostaining followed by a flow cytometric analysis showing expression of galectin-3 in AGS and MKN45 cells. An isotype control is also shown. The data are shown as the mean fluorescence intensity (MFI) obtained from three independent experiments. ***p < 0.001 compared to MKN45.

The molecular mechanisms of galectin-3 upregulation remain unclear. Consistent with the finding that galectin-3 expression increases in gastric cancers [38], the expression of galectin-3 was higher in AGS cells as demonstrated by Western blotting (Figure 4A) and an immunostaining-based flow cytometric analysis (Figure 4B). Compared to MKN45 cells, the increased galectin-3 expression in AGS cells was correlated with constitutive activation of PI3K (by detecting PIP3 generation) and AKT and a decrease in PTEN expression (Figure 4A). To determine the potential mechanisms for the increase in galectin-3, the roles of PI3K activation and decreased PTEN expression were pharmacologically determined by inhibiting PI3K and genetically restoring PTEN expression. However, neither LY294002 treatment (Figure 4C) nor exogenous PTEN expression restored (Figure 4D) the changes in galectin-3 expression in AGS cells, although these treatments efficiently reduced PIP3 generation and AKT activation. These results show a PI3K- and PTEN-independent increase in galectin-3 expression in AGS cells, potentially leading to AKT activation.

### Changing galectin-3 expression does not alter PI3K activity but changes AKT phosphorylation at the threonine residue

**Figure 5:**
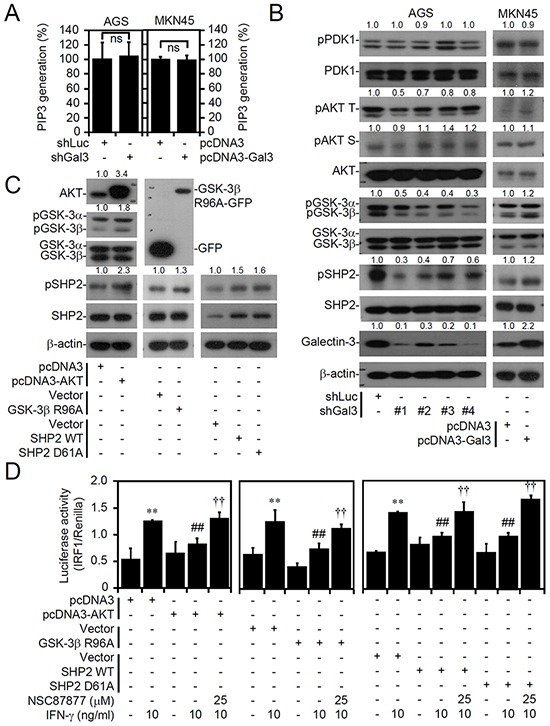
Galectin-3 facilitates activation of the AKT/GSK-3β/SHP2 signaling pathway downstream of PI3K and PDK1, and overexpression of AKT, inactive GSK-3β, and SHP2 suppress interferon (IFN)-γ-activated IRF1 A PIP3 MASS ELISA Kit assay and Western blot analysis showing PI3K activity **A.** and expressions of the indicated proteins **B.** in AGS cells (IFN-γ insensitive) transfected with shRNA targeting luciferase (*shLuc*) and shRNA targeting galectin-3 (*shGal3*; clones 1-4) and MKN45 cells (IFN-γ sensitive) transfected with pcDNA3- and pcDNA3-Gal3. For kinase activity, the data are presented as the mean ± SD from three independent experiments. ns, not significant. **C.** Representative Western blot of the indicated proteins in pcDNA3-, pcDNA3-AKT-, GFP-, GFP-tagged GSK-3β^R96A^-, pBABE-, pBABE-SHP2 wild-type-, and pBABE-SHP2^D61A^-tranfected MKN45 cells (IFN-γ sensitive). For Western blotting, β-actin was used as an internal control. pAKT T, phospho-AKT at Thr308; pAKT S, phospho-AKT at Ser473. A representative dataset from triplicate experiments is shown. **D.** In cells transfected with or without treatment with IFN-γ or the SHP2 inhibitor, NSC87877, for 6 h, the luciferase reporter assay showed the ratio of IRF1 to control Renilla. The data are presented as the mean ± SD from three independent experiments. **p < 0.01 and ***p < 0.001 compared to the untreated sample; ^##^*p* < 0.01 and ^###^*p* < 0.001 compared to the relative control. ^††^*p* < 0.01 and ^†††^*p* < 0.001 compared to IFN-γ.

To verify the potential effects of galectin-3 on AKT, we next investigated the potential role of galectin-3 in regulating PI3K, as previous studies reported that galectin-3 acts upstream of PI3K/AKT/GSK-3β signaling [12, 14]. However, neither galectin-3 silencing in AGS cells nor galectin-3 overexpression in MKN45 cells produced any changes in PI3K activities (Figure 5A), indicating an independent role for galectin-3 in PI3K activation. Interestingly, decreasing galectin-3 using shRNA or increasing galectin-3 by overexpression both significantly caused changes in AKT phosphorylation at Thr308, without affecting PDK1, an upstream kinase that specifically phosphorylates AKT at Thr308 (Figure 5B). Furthermore, silencing galectin-3 in AGS cells induced decreases in GSK-3β and SHP2 phosphorylation at Ser9 and Thr542, respectively, while galectin-3 overexpression in MKN45 cells induced the opposite effects (Figure 5B). In addition, the galectin-3-silenced human myeloid leukemia cell lines THP1 and U937 were used to confirm the identified effects (Supplementary Figure S4). These findings demonstrate potential regulation of AKT/GSK-3β/SHP2 signaling by galectin-3, independent of direct effects on PI3K and PDK1.

To address the induction of SHP2-induced cellular unresponsiveness to IFN-γ by AKT/GSK-3β signaling, we overexpressed AKT, GSK-3β^R96A^ (inactive GSK-3β), SHP2, and SHP2^D61A^ (active SHP2) in MKN45 cells to evaluate the cellular unresponsiveness to IFN-γ stimulation. Protein expression was assessed by Western blot analysis, and the results confirmed the activation of AKT/GSK-3β/SHP2 signaling through AKT overexpression, GSK-3β inactivation, and SHP2 activation, as AKT, GSK-3β, and SHP2 were phosphorylated at Ser473, Ser9, and Tyr542, respectively (Figure 5C). Following IFN-γ stimulation with or without SHP2 inhibition using the selective inhibitor NSC87877, we observed that AKT-, GSK-3β^R96A^-, SHP2-, and SHP2^D61A^-transfected MKN45 cells were significantly resistant to IFN-γ-induced STAT1 phosphorylation at Tyr701 (Supplementary Figure S5) and IRF1 transactivation (Figure 5D) in a SHP2-dependent manner. However, changes in IFN-γ receptor 1 (IFNGR1) and IFNGR2 expression levels were observed in the transfected MKN45 cells (Supplementary Figure S6). These results confirm the finding that the AKT/GSK-3β/SHP2 signaling axis induces cellular unresponsiveness to IFN-γ signaling.

### Decreased galectin-3 facilitates IFN-γ-induced cell growth inhibition and apoptosis in AGS cells

**Figure 6:**
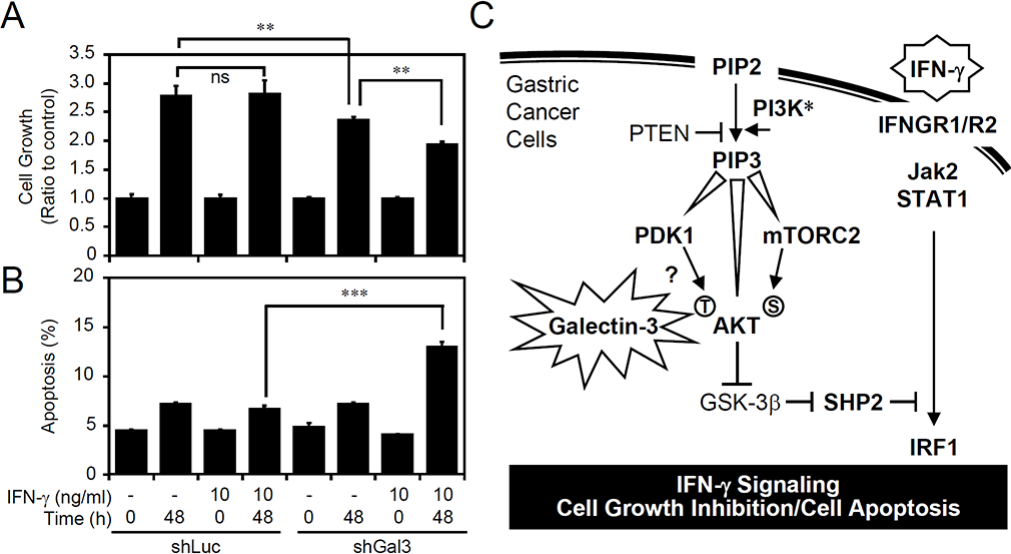
Silencing galecin-3 sensitizes AGS cells to interferon (IFN)-γ-induced cell growth inhibition and apoptosis A proliferation assay **A.** and propidium iodide (PI) staining-based flow cytometric analysis **B.** showing cell growth and apoptosis, respectively, in IFN-γ-treated AGS cells transfected with shRNA targeting luciferase (*shLuc*) and shRNA targeting galectin-3 (*shGal3*). The data (mean ± SD from three independent experiments) are shown as multiples of change compared to the normalized value of the control sample. **p < 0.01 and ***p < 0.001. ns, not significant. **C.** A hypothetical model for aberrant galectin-3 involved in facilitating AKT/GSK-3β/SHP2 signaling to inhibit IFN-γ STAT1/IRF1 signaling and IFN-γ-induced cell growth inhibition and apoptosis.

We showed that galectin-3 is vital for AKT/GSK-3β/SHP2-induced IFN-γ resistance in AGS cells. We therefore hypothesized that galectin-3 contributes to the growth of AGS cells and to the generation of resistance to IFN-γ-induced cell growth inhibition and apoptosis. Consistent with the results of previous studies showing that galectin-3 is required for gastric cancer cell growth [38], galectin-3 silencing significantly inhibited the growth of AGS cells (Figure 6A). Indeed, in IFN-γ-insensitive AGS cells, cell growth was not inhibited by IFN-γ treatment as previously demonstrated [35]; however, silencing galectin-3 expression ultimately significantly facilitated IFN-γ-induced cell growth inhibition (Figure 6A) and cell apoptosis (Figure 6B). These findings demonstrated that an increase in galectin-3 induces cellular unresponsiveness to IFN-γ-induced growth inhibition and cell apoptosis.

## DISCUSSION

No studies have demonstrated the intracellular role of galectin-3 in regulating IFN-γ signaling and bioactivity. IFN-γ treatment might increase or decrease galectin-3 expression [39, 40], suggesting that galectin-3 is a potential regulator of IFN-γ signaling as a homeostatic mechanism. However, in melanoma cells, IFN-γ stimulation causes galectin-3 downregulation followed by cell growth inhibition [39]. To the best of our knowledge, the present study is the first to show an inhibitory effect of intracellular galectin-3 on IFN-γ signaling. Although increased galectin-3 has been well-known for a correlation with AKT activation, as summarized in Figure 6C, an increase in galectin-3 might facilitate PDK1-induced AKT phosphorylation at threonine residues to deactivate GSK-3β. Additionally, upregulated galectin-3 does not enhance AKT phosphorylation at serine residues, while mTORC2 determines this phosphorylation. Consistent with previous studies suggesting that GSK-3β potentially inhibits SHP2 to facilitate IFN-γ signaling [34, 35], galectin-3 stimulates the identified GSK-3β/SHP2 pathway by activating AKT. Regarding a potential role of galectin-3 in promoting tumorigenesis, it is hypothesized that galectin-3 may decrease IFN-g signaling by facilitating AKT/GSK-3b/SHP2 signaling.

Mechanisms for galectin-3 overexpression in cancer cells remain unclear, although several transcription factors, such as Sp1, cAMP response element-binding protein, nuclear factor (NF)-κB, activator protein (AP)-1, Runx2, and Ras/mitogen-activated protein kinase (MAPK) signaling, have been suggested to be involved [8], while p53 shows negative regulation of galectin-3 [41, 42]. The involvement of p53 is precluded because both AGS and MKN45 cells are wild-type p53-bearing gastric cancer cells. We previously showed that activation of NF-κB and Ras/MAPK promoted AGS cell hyperproliferation and migration [43]. The potential roles of NF-κB and Ras/MAPK in inducing galectin-3 expression require further investigation.

Galectin-3 is expressed in nuclei, cytoplasm, mitochondria, extracellular spaces and on cell surfaces [8]. The extracellular role of galectin-3 in AGS cells is precluded in the present study, as inhibiting galectin-3 through the exogenous administration of lactose neither decreased AKT nor reversed IFN-γ resistance. Expression of galectin-3 is increased in primary gastric cancer and metastatic lymph nodes [11]. Galectin-3 facilitates cell survival against apoptosis and cell growth inhibition induced by the tumor necrosis factor-related apoptosis-inducing ligand, Adriamycin, staurosporine, and cisplatin [14, 15, 36, 44]]. With a cisplatin-induced galectin-3 increase, we also observed that galectin-3-expressing cells were more resistant to IFN-γ signaling. In addition to enhancing survival responses, chemotherapeutic-resistant cells showed antiapoptotic properties and IFN-γ resistance via a mechanism involving galectin-3 induction. This requires further demonstration by decreasing galectin-3.

Compared to galectin-3-deficient cancers, galectin-3-positive cancers are correlated with the presence of inactive GSK-3β, which might facilitate the Wnt/β-catenin pathway [45]. Regarding the negative regulation of galectin-3 on GSK-3β-activated IFN-γ signaling, it is important to verify the effects of galectin-3 on PI3K/AKT signaling, which is important for GSK-3β inactivation. Elad-Sfadia and colleagues [46] originally demonstrated that galectin-3 is required for Ras-induced PI3K/AKT activation in response to growth factor stimulation. Some cancer cells possessing galectin-3 advancement showed enhanced PI3K/AKT signaling [12, 14]. Although we showed that increasing or decreasing galectin-3 expression did not interfere with the activity of PI3K, these results were inconsistent, potentially reflecting: (1) the inability of Ras to induce PI3K/AKT activation in AGS cells as previously demonstrated [43]; (2) the attenuation of PIP3-based AKT activation through PI3K inhibition, even with galectin-3 overexpression [12, 14]; and (3) different responses in different cell types causing differences in the regulation of galectin-3/PI3K/AKT signaling.

There is no evidence for showing an interaction between galectin-3 and AKT, although galectin-3 benefits AKT activation [8]. In addition to PI3K, upstream signaling molecules, such as PDK1, ILK, and mammalian target of rapamycin (mTOR), should therefore be further investigated. Accordingly, we showed that changing galectin-3 expression altered AKT phosphorylation at threonine residues. Without increasing PI3K activity, galectin-3 acted upstream of AKT and downstream of PDK1, while PDK1 specifically phosphorylated AKT at threonine residues. It is commonly demonstrated that galectin-3 function extracellularly; however, galectin-3 can be expressed within the cells and may function as an intracellular regulator. The underlying mechanism of galectin-3-induced AKT activation remains undefined; however, our results suggested that an intracellular galectin-3 may act as an essential regulator for facilitating PDK1-mediated AKT phosphorylation. Further studies are needed to clarify the intracellular role of galectin-3.

SHP2 is overexpressed and hyperactivated in gastric tumors [5, 47–49]. However, regulation of the expression and activation of SHP2 remains unclear. We previously showed the upstream role of GSK-3β in SHP2 inhibition [34], and, in gastric AGS cells, PI3K/AKT-induced GSK-3β inactivation might trigger SHP2 activation for IFN-γ resistance [35]. In the present study, we demonstrated that increased galectin-3 expression facilitates PI3K-induced AKT/GSK-3β/SHP2 pathway activation, thereby inducing cellular unresponsiveness to IFN-γ. These findings support the idea that deactivating GSK-3β promotes SHP2 activation, which inhibits IFN-γ signaling and bioactivities, such as cancer cell growth inhibition and apoptosis. Regarding the anticancer properties of IFN-γ [7, 50, 51], the implication of PI3K/PTEN/galectin-3/AKT/GSK-3β/SHP2 signaling might reflect a strategy hijacked by cancer cells to cause cellular unresponsiveness to IFN-γ. Ablating IFN-γ resistance by inhibiting the pathway identified herein and in previous studies [35] may provide benefits for treating IFN-γ-unresponsive gastric cancers.

## MATERIALS AND METHODS

### Cell cultures and reagents

Human primary AGS (CRL-1739, ATCC) and metastatic MKN45 (JCRB0254, The RIKEN Cell Bank, Koyadai, Japan) gastric adenocarcinoma cells were routinely grown on plastic plates in F-12 nutrient mixture (Ham) and RPMI medium 1640 (F-12, RPMI; Invitrogen Life Technologies, Rockville, MD), respectively, with L-glutamine and 15 mM HEPES supplemented with 10% heat-inactivated fetal bovine serum (FBS), 50 units of penicillin, and 50 μg/ml of streptomycin and were maintained in a humidified atmosphere of 5% CO_2_ and 95% air. Cells were suspended in trypsin/EDTA and counted. The reagents and antibodies used were PI3K inhibitor 2-(4-morpholinyl)-8-phenyl-4H-1-benzopyran-4-one hydrochloride (LY294002), cisplatin, lactose, and dimethyl sulfoxide (DMSO) (Sigma-Aldrich, St. Louis, MO); recombinant human IFN-γ (PeproTech, Rocky Hill, NJ); anti-GSK-3α/β and anti-GFP (Santa Cruz Biotechnology, Santa Cruz, CA); antibodies against phospho-STAT1α/β at Tyr701, STAT1α/β, phospho-AKT at Thr308 and Ser473, AKT, phospho-SHP2 at Tyr542, SHP2, phospho-GSK-3α/β at Ser21/9, PTEN, phospho-PDK1 Ser241, and PDK1 (Cell Signaling Technology, Beverly, MA); antibodies against galectin-3 (Abcam, Cambridge, MA); a mouse monoclonal antibody specific for β-actin, histone, and tubulin (Chemicon International, Temecula, CA); and Alexa Fluor 488- and horseradish peroxidase (HRP)-conjugated goat anti-mouse, goat anti-rabbit, and donkey anti-goat immunoglobulin G (IgG) (Invitrogen, Carlsbad, CA). All drug treatments were assessed for cytotoxic effects using cytotoxicity assays prior to the experiments. Non-cytotoxic dosages were used in this study.

### Plasmid transfection

Transient transfection was performed using an MP-100 Microporator (Digital BioTechnology, Seoul, Korea) according to the manufacturer's instructions for optimization and usage. The pcDNA 3.1-green fluorescent protein (GFP) constructs expressing a constitutively inactive form of GSK-3β^R96A^ were kindly provided by Dr. Pei-Jung Lu (Institute of Clinical Medicine, College of Medicine, National Cheng Kung University, Tainan, Taiwan). The plasmid expressing GFP-PTEN (ID NM_000314; Plasmid 13039) and its control pcDNA3-GFP (Plasmid 13031); pcDNA3 flag HA AKT1 (Plasmid 9021) and its control pcDNA3 flag HA (Plasmid 1436); and pBABE-puro SHP2 (Plasmid 8329) and pBABE-puro SHP2^D61A^ (Plasmid 8330) and their control vector pBABE-puro (Plasmid 1764) were purchased from Addgene (Cambridge, MA). After transfection, cells were cultured for 24 h before the experiments.

### Western blotting

Harvested cells were lysed in buffer containing 1% Triton X-100, 50 mM Tris (pH 7.5), 10 mM EDTA, 0.02% NaN_3_, and a protease inhibitor cocktail (Roche Boehringer Mannheim Diagnostics, Mannheim, Germany). After freeze-thawing, cell lysates were centrifuged at 10^4^ × *g* and 4°C for 20 min. For the cytosolic/nuclear protein analysis, protein fractions were isolated using a Compartmental Protein Extraction Kit (Calbiochem, San Diego, CA) according to the manufacturer's instructions. The lysates and supernatants were boiled in sample buffer for 5 min. The proteins were subsequently subjected to sodium dodecylsulfate polyacrylamide gel electrophoresis (SDS-PAGE) and transferred to polyvinylidene difluoride membranes (Millipore, Billerica, MA) using a semi-dry electroblotting system. After blocking with 5% skim milk in phosphate-buffered saline (PBS), the membranes were incubated overnight with a 1:1000 dilution of primary antibodies at 4°C. The membranes were subsequently washed with 0.05% PBS-Tween 20 and incubated with a 1:5000 dilution of an HRP-conjugated secondary antibody at room temperature for 1 h. After washing, membranes were soaked in an enhanced chemiluminescence (ECL) solution (PerkinElmer Life and Analytical Sciences, Boston, MA) for 1 min and exposed to x-ray film (BioMax; Eastman Kodak, Rochester, NY). The relative signal intensity was quantified using ImageJ software (version 1.41o; W. Rasband, National Institutes of Health, Bethesda, MD). Changes in the ratio of proteins compared to the normalized value of untreated cells (indicated protein/β-actin or phosphorylated protein/total protein/β-actin) were also determined. One set of representative data obtained from three independent experiments is shown.

### Luciferase reporter assay

For the luciferase reporter assay, cells were transiently co-transfected, using GeneJammer reagent (Stratagene, La Jolla, CA), with an IRF1 promoter-driven luciferase reporter (0.2 μg) and 0.01 μg of *Renilla* luciferase-expressing plasmid (pRL-TK; Promega, Madison, WI). Twenty-four hours post-transfection, cells were treated with IFN-γ for 6 h, lysed, and subsequently harvested for luciferase and *Renilla* measurements using a luciferase assay system (Dual-Glo; Promega). For each lysate, the firefly luciferase activity was normalized to *Renilla* luciferase activity to assess transfection efficiencies.

### PI3K activity assay

A PIP3 mass enzyme-linked immunosorbent assay (ELISA; K-2500s, Echelon Biosciences, Salt Lake City, UT) was performed to detect PI3K activity in cells according to the manufacturer's instructions.

### Immunostaining

To detect galectin-3 expression, we fixed, stained, and analyzed cells. For the flow cytometric analysis, cells were stained with anti-galectin-3 antibodies, followed by incubation with a mixture of Alexa Fluor 488-conjugated goat anti-rabbit IgG. Cells were analyzed using flow cytometry (FACSCalibur; BD Biosciences, San Jose, CA) with excitation at 488 nm; emission was detected with the FL-1 channel (at 515∼545 nm). Samples were analyzed using CellQuest Pro 4.0.2 software (BD Biosciences), and quantification was performed using WinMDI 2.8 software (The Scripps Institute, La Jolla, CA). Small cell debris was excluded by gating on a forward scatter plot. After washing twice with PBS, tissue sections were incubated with primary antibodies in antibody diluents (DAKO, Carpentaria, CA) at 4°C overnight.

### RNA interference

Protein expression was downregulated using lentiviral expression of short hairpin (sh)RNA targeting galectin-3 (Clone1, TRCN0000029304 containing the following shRNA target sequence: 5′-GCTCACTTGTTGCAGTACAAT-3′; Clone2, TRCN0000029306 containing the following shRNA target sequence: 5′-GCAAACAGAATTGCTTTAGAT-3′; Clone3, TRCN0000029307 containing the following shRNA target sequence: 5′-GCAGTACAATCATCGGGTTAA-3′; and Clone4, TRCN0000029308 containing the following shRNA target sequence: 5′-GCAATACAAAGCTGGATAAT-3′) and a negative control construct (luciferase shRNA, shLuc). shRNA clones were obtained from the National RNAi Core Facility, Institute of Molecular Biology/Genomic Research Center, Academia Sinica, Taipei, Taiwan. Lentiviruses were prepared, and cells were infected according to previously described protocols [52]. Briefly, AGS cells were transduced using a lentivirus with an appropriate multiplicity of infection in complete growth medium supplemented with polybrene (Sigma-Aldrich). After transduction for 24 h and puromycin (Calbiochem, San Diego, CA) selection for 3 days, protein expression was monitored using a Western blot analysis.

### Cell growth assay

To measure cell growth, cell viability was determined using a colorimetric assay (Cell Counting Kit-8; Dojindo Molecular Technologies, Kumamoto, Japan) according to the manufacturer's instructions. A microplate reader (SpectraMax 340PC; Molecular Devices, Sunnyvale, CA) was used to measure the absorbance at 450 nm, and data were analyzed using Softmax Pro software (Molecular Devices). The relative growth rate was normalized to the control group.

### Cell apoptosis assay

Cell apoptosis was analyzed using propidium iodide (PI; Sigma-Aldrich) staining with RNase reaction and then analyzed using flow cytometry (FACSCalibur; BD Biosciences) with excitation at 488 nm. Samples were analyzed using WinMDI 2.8 software (The Scripps Institute). Apoptotic cells were gated and quantified in the sub-G_1_ phase.

### Statistical analysis

Values are expressed as the mean ± standard deviation (SD). Significant differences between groups were assessed using a one-way analysis of variance (ANOVA), followed by Dunnett's post-hoc test, Student's *t* test, or an ANOVA, as appropriate. These analyses were performed using GraphPad Prism 4 software (GraphPad Software, La Jolla, CA). Exact *p* values are listed in the corresponding figure legends. Statistical significance was set at *p* < 0.05.

## SUPPLEMENTARY FIGURES


