# Supplementary material for: An increase in galectin-3 causes cellular unresponsiveness to IFN-γ-induced signal transduction and growth inhibition in gastric cancer cells
Source: Oncotarget. 2016 Feb 26;7(12):15150–60. doi: 10.18632/oncotarget.7750 (PMC4924776; doi:10.18632/oncotarget.7750)
Supplement: Supplementary file 1 [file oncotarget-07-15150-s001.pdf]

SUPPLEMENTARY FIGURES

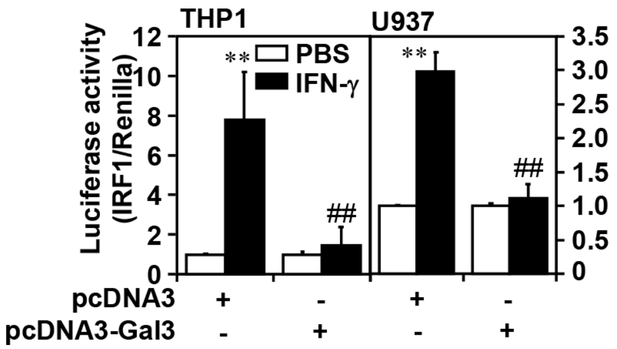

Supplementary Figure S1: Overexpression of galectin-3 inhibits IFN-γ-activated IRF1. A luciferase reporter assay was used to detect IRF1 transactivation in IFN-γ-treated pcDNA3- and pcDNA3-Gal3-transfected THP1 and U937 cells. Data are the means ± SD from three independent experiments. $^{**}P < 0.01$ compared with PBS; $^{\#\#}P < 0.01$ compared with relative control.

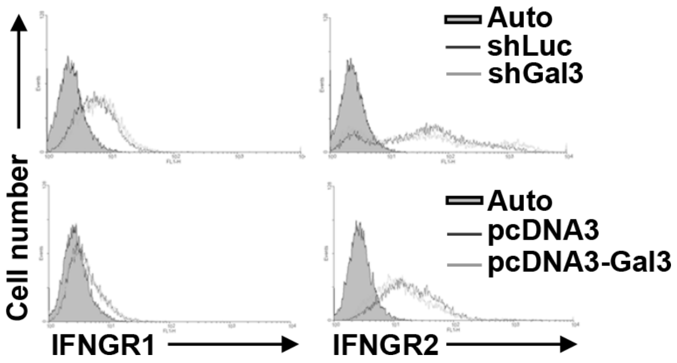

Supplementary Figure S2: No changes on IFNGRs in galectin-3-silencing and/or galectin-3-overexpressing cells. Immunostaining followed by flow cytometric analysis was used to detect IFNGR1 and IFNGR2 expression in shRNA targeting luciferase (*shLuc*)- and shRNA targeting galectin-3 (*shGal3*)-transfected AGS cells and pcDNA3- and pcDNA3-Gal3-transfected MKN45 cells. An isotype control was also shown. A representative data set from triplicate experiments is shown.

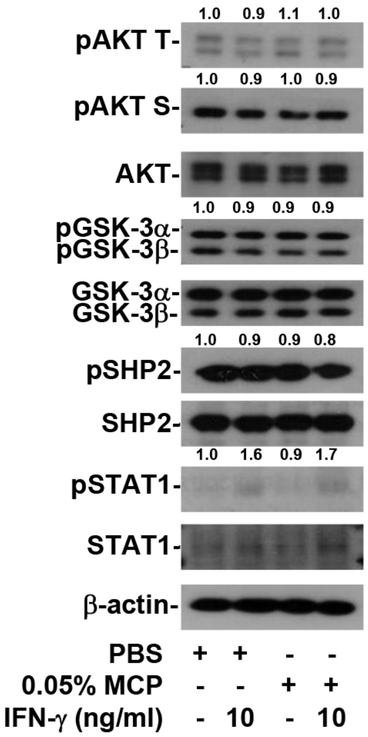

Supplementary Figure S3: No changes on AKT/GSK-3β/SHP2 and IFN-γ-activated STAT1 in modified citrus pectin (MCP)-treating AGS cells. Western blots of the indicated proteins in 0.05% MCP-treated AGS cells with or without IFN-γ stimulation for 1 h. β-actin was used as an internal control. A representative data set from triplicate experiments is shown.

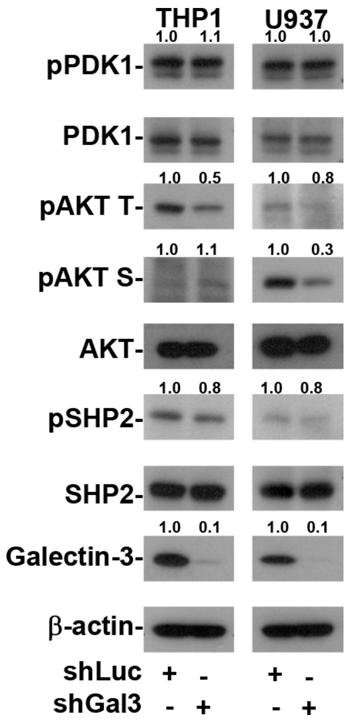

Supplementary Figure S4: Galectin-3 regulates AKT/SHP2 signaling in THP1 and U937 myeloid leukemia cells. Western blots of the indicated proteins in shRNA targeting luciferase (*shLuc*)- and shRNA targeting galectin-3 (*shGal3*)-transfected THP1 and U937 cells. β-actin was used as an internal control. A representative data set from triplicate experiments is shown.

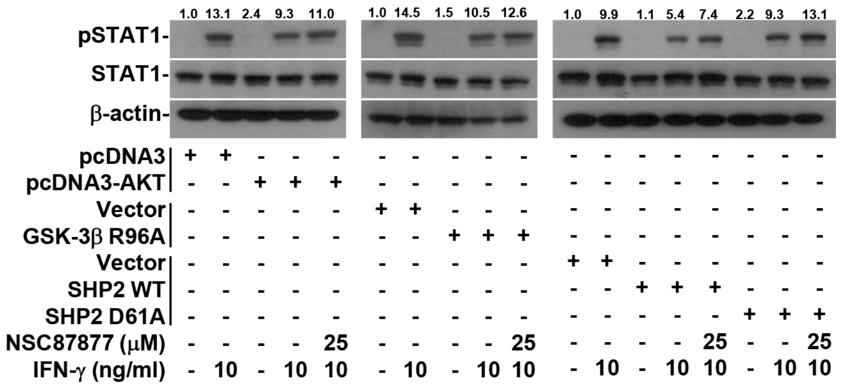

Supplementary Figure S5: Inhibiting SHP2 reverses STAT1 phosphorylation in AKT-, GSK-3β^{R96A}-, and SHP2-overexpressing MKN45 cells. Following IFN-γ treatment (6 h post-treatment) in the presence of SHP2 inhibitor NSC87877 (0.5 h pre-treatment), Western blot showed phosphorylated STAT1 Tyr701 (pSTAT1) and STAT1 in pcDNA3-, pcDNA3-AKT-, GFP-, GFP-tagged GSK-3β^{R96A}-, pBABE-, pBABE-SHP2 wild-type-, and pBABE-SHP2^{D61A}-tranfected MKN45 cells (IFN-γ sensitive). β-actin was used as an internal control.

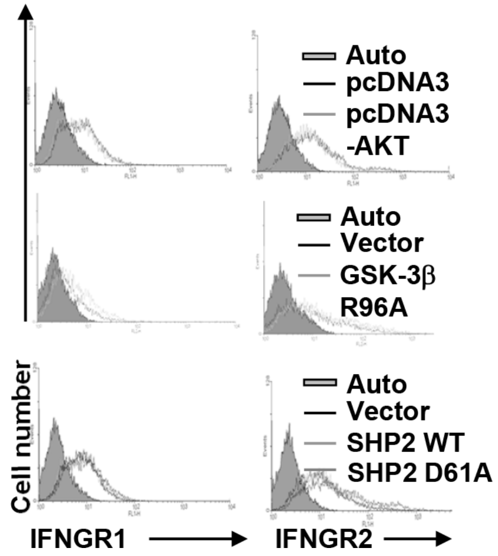

Supplementary Figure S6: Expression of IFNGR1 and IFNGR2 in AKT-, GSK-3β^{R96A}-, and SHP2-overexpressing MKN45 cells. Immunostaining followed by flow cytometric analysis was used to detect IFNGR1 and IFNGR2 expression in pcDNA3-, pcDNA3-AKT-, GFP-, GFP-tagged GSK-3β^{R96A}-, pBABE-, pBABE-SHP2 wild-type-, and pBABE-SHP2^{D61A}-tranfected MKN45 cells. An isotype control was also shown. A representative data set from triplicate experiments is shown.
